# Performance of the FreeStyle Libre Flash Glucose Monitoring System during an Oral Glucose Tolerance Test and Exercise in Healthy Adolescents

**DOI:** 10.3390/s23094249

**Published:** 2023-04-25

**Authors:** Sahar Afeef, Keith Tolfrey, Julia K. Zakrzewski-Fruer, Laura A. Barrett

**Affiliations:** 1 School of Sport, Exercise and Health Sciences, Loughborough University, Loughborough LE11 3TU, UK; 2 Clinical Nutrition Department, Faculty of Applied Medical Sciences, King Abdulaziz University, Jeddah 21589, Saudi Arabia; 3 Institute for Sport and Physical Activity Research, University of Bedfordshire, Bedford MK41 9EA, UK

**Keywords:** accuracy, flash glucose monitor, continuous glucose monitor, interstitial glucose, capillary glucose, postprandial glucose, OGTT, exercise, MARD, healthy adolescents

## Abstract

This study’s aim was to assess FreeStyle Libre Flash glucose monitoring (FGM) performance during an oral glucose tolerance test (OGTT) and treadmill exercise in healthy adolescents. This should advance the feasibility and utility of user-friendly technologies for metabolic assessments in adolescents. Seventeen healthy adolescents (nine girls aged 12.8 ± 0.9 years) performed an OGTT and submaximal and maximal treadmill exercise tests in a laboratory setting. The scanned interstitial fluid glucose concentration ([ISFG]) obtained by FGM was compared against finger-prick capillary plasma glucose concentration ([CPG]) at 0 (pre-OGTT), −15, −30, −60, −120 min post-OGTT, pre-, mid-, post- submaximal exercise, and pre- and post- maximal exercise. Overall mean absolute relative difference (MARD) was 13.1 ± 8.5%, and 68% (*n* = 113) of the paired glucose data met the ISO 15197:2013 criteria. For clinical accuracy, 84% and 16% of FGM readings were within zones A and B in the Consensus Error Grid (CEG), respectively, which met the ISO 15197:2013 criteria of having at least 99% of results within these zones. Scanned [ISFG] were statistically lower than [CPG] at 15 (−1.16 mmol∙L^−1^, *p* < 0.001) and 30 min (−0.74 mmol∙L^−1^, *p* = 0.041) post-OGTT. Yet, post-OGTT glycaemic responses assessed by total and incremental areas under the curve (AUCs) were not significantly different, with trivial to small effect sizes (*p* ≥ 0.084, d = 0.14–0.45). Further, [ISFGs] were not different from [CPGs] during submaximal and maximal exercise tests (interaction *p* ≥ 0.614). FGM can be a feasible alternative to reflect postprandial glycaemia (AUCs) in healthy adolescents who may not endure repeated finger pricks.

## 1. Introduction

Postprandial glycaemia has been implicated in developing cardiometabolic diseases [1]. Even in healthy individuals with normoglycaemia, those with a greater glycaemic response after feeding have reduced insulin sensitivity and impaired beta-cell function and, therefore, are at higher risk of developing type 2 diabetes (T2D) and cardiovascular disease (CVD) [2,3,4]. Thus, moderating postprandial glycaemia through dietary manipulation and physical activity is essential for disease prevention. With the increasing prevalence of prediabetes in the UK population (aged 16 to 39 years) from 2.8% in 2003 to 15.6% in 2011 [5] and 2.4 million people (aged 17 or greater) in England recorded by GP practices as having non-diabetic hyperglycaemia for the period January 2020 to March 2021 [6], devices such as continuous glucose monitoring (CGM) systems have become of greater importance for research purposes. Researchers have utilised CGM in healthy adolescents without diabetes to understand the influence of lifestyle behaviours such as dietary intake and physical activity on glycaemic responses [7,8]. Yet, the validity of CGM has not been well-established in this sub-group of the population [9]. One known limitation of the use of CGM devices is a time delay due to the diffusion processes of glucose from the vascular to the interstitial space, which is a particular problem when glucose concentrations are changing rapidly (e.g., during high intensity exercise or when foods containing high amounts of glucose are ingested). Although the time lag of glucose from the intravascular to the interstitial compartments has been investigated in adults [10], this has yet to be investigated in healthy adolescents and could be different, particularly during exercise when substrate utilisation is known to be different in adults due to metabolic and hormonal differences [11]. Thus, it is important to validate CGM, as it would inform preventative approaches for cardiometabolic disease and ultimately help to improve the health outcomes of this vulnerable population.

The Flash glucose monitor (FGM; FreeStyle Libre, Abbott Diabetes Care Inc.), a type of CGM system, has the advantage of being less invasive than the commonly used method of finger-prick blood sampling in paediatric research. Indeed, FGM has been shown to be less painful and highly acceptable over a finger-brick technique using a glucometer among adolescents with type 1 diabetes (T1D) [12,13]. However, it must be acknowledged that the cost of the devices can be prohibitive for some users, and issues with the insertion, adhesion and removal of sensors have been reported [14]. The FGM is inserted once into the back of the upper arm to measure interstitial fluid glucose concentration ([ISFG]) via a glucose oxidase reaction, and it uses algorithms to estimate blood glucose concentration [15]. Furthermore, the FGM does not require user calibration (i.e., it is factory-calibrated) using a glucometer, which is advantageous for healthy adolescents who are unfamiliar with this technique. The FGM provides two types of data: scanned glucose (manual) and historic glucose (automatic). The patients/participants can view the scanned glucose concentrations on the reader following manual sensor scanning. The historic glucose concentrations are recorded every 15 min and require active (manual) scanning of the sensor every 8 h to store the data and avoid its loss. Thus, the FGM can provide broader information on glycaemic variability under free-living and laboratory conditions. Such information would not be detected using the traditional finger-prick method, which is not appropriate for use over extended durations with such high sampling frequencies. This is important from a health perspective because glycaemic variability is recognised as a crucial component of glycaemic control [16] associated with increased CVD risk [4,17]. For this reason, comparing FGM with the traditional finger-prick method is needed in healthy adolescents without diabetes to facilitate its application in research.

The FGM has been shown to be safe and have acceptable accuracy (based on overall mean absolute relative difference (MARD); 11.7–13.9%) in young people with diabetes; yet, its performance deteriorated at low glucose concentrations and when the rate of glucose change was high [12,18,19]. There is a lack of information on the ability of FGM to detect glycaemic responses to feeding and exercise in healthy adolescents. To our knowledge, only a few studies have examined the validity of FreeStyle Libre in response to feeding and exercise in adults without diabetes [20,21,22,23] with only a single study validating the FreeStyle Libre Pro version in healthy children aged 7 to 12 years [9]. One study showed that FGM overestimated venous plasma glucose concentrations by 0.63 to 1.50 mmol∙L^−1^ 30 to 90 min after glucose loading in a small sample (*n* = 7) of healthy adults [23]. However, two recent studies demonstrated that while FGM tended to underestimate plasma glucose concentrations by 1.03 to 1.61 mmol∙L^−1^ in response to a standard breakfast meal, the overall accuracy of FGM was deemed clinically acceptable in healthy adults [20,21]. It is difficult to identify the reason for such contrasting results, but it could be partly due to the differences in test meals and/or participant characteristics. Physical activity can also impact glucose concentrations, yet the accuracy of FGM during different exercise intensities does not appear to have been determined in healthy adolescents. Two recent studies with healthy adults showed conflicting results of FGM performance during exercise. One study with healthy adults showed a reduced accuracy of FGM during high intensity intermittent exercise, as indicated by high values in the clinically unsafe zone (i.e., 10.5% in zone D) after consuming a carbohydrate-rich meal [22]. Another study showed FGM underestimated plasma glucose concentrations during different walking conditions, with 99.6 to 100% of glucose values within the clinically acceptable zones A and B [21]. Yet, findings based on adults may not apply to adolescents’ distinct hormonal and metabolic profiles [11]. Therefore, the aim of this study was to compare blood glucose concentrations obtained by FGM (i.e., [ISFG]) against [CPG] (reference method) in response to an oral glucose tolerance test (OGTT) and treadmill exercise at different intensities in a laboratory setting in healthy adolescents to assess the degree of measurement bias.

## 2. Materials and Methods

This was a prospective, single-arm study performed in healthy adolescents. The study was conducted between November 2019 and March 2020 in accordance with the ethical standards of Loughborough University Ethics Committee (HPSC reference number: R19-P147). The ethical approval was obtained in October 2019. Participants were recruited through school assemblies, where the aims and procedures of the study were presented. Written assent was obtained from each participant, and written informed consent was obtained from a parent/guardian.

### 2.1. Eligibility Criteria

The participants were included providing they were aged 11–14 years, not on medication or living with a disease (e.g., diabetes) that may affect glucose metabolism, not presenting with any injuries or conditions that prevented them from performing any exercise task (e.g., musculoskeletal problems, epilepsy, uncontrolled exercise-induced asthma), and had no skin conditions (e.g., allergy) that may affect glucose sensor employment.

### 2.2. Study Design

An FGM (FreeStyle Libre, Abbott Diabetes Care Inc., UK) was inserted in the participant’s non-dominant upper arm according to the manufacturer’s instructions after the skin was cleaned with an alcohol swab, and it was worn for 14 days. An adhesive patch (Hypafix^®^ transparent, BSN medical) was placed over the glucose monitor to ensure it was attached securely, and the participants were given extra patches to replace those that became dirty or lost adhesion. To avoid the reported inaccuracy of the monitor on the insertion day [24], the monitor was validated against finger-prick [CPG] on day 2 or 3 of sensor wear in response to feeding and exercise. Participants were asked to refrain from engaging in any physical activity of moderate–vigorous intensity 24 h preceding the laboratory visit. Additionally, participants were asked to consume a standardised cereal bar at 19:20, after which they were asked to drink only water to ensure they had fasted for 12 h when they arrived at the lab the following day.

Participants arrived at the laboratory wearing an FGM, and each time a capillary blood sample was taken, the FGM was scanned manually with the reader device by a researcher to obtain simultaneous glucose readings from the two sites. Anthropometry was conducted first, and then participants rested for 10 min before providing a fasting capillary blood sample. Then, they were asked to consume a drink containing 1.75 g glucose per kg body mass (with a maximum of 75 g of glucose in line with standard OGTT procedure) within 5 min [25]. Subsequent capillary blood samples were collected at 15-, 30-, 60-, and 120-min intervals after the glucose drink consumption was initiated. After completing the OGTT, participants were given a breakfast meal to satisfy their hunger (white bread, butter, jam, and fruit juice, which provided 422 kcal (1766 kJ); 73% carbohydrate; 16% protein; 8% fat).

**Figure 1:**
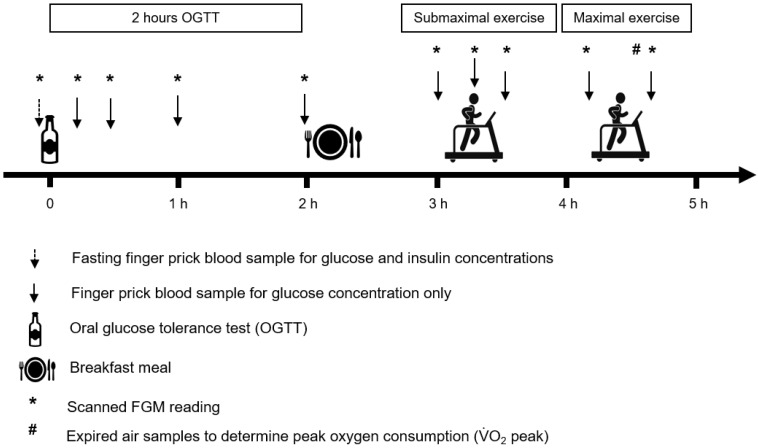
Schematic of the study protocol.

One hour after breakfast consumption, the participants completed a 4 × 4 min stage submaximal treadmill exercise test. The treadmill speed was increased after each four-minute stage: 4, 6, 8 and 10 km·h^−1^ for boys and 4, 5, 6.5, 8 km·h^−1^ for girls. The first two stages equated to walking speeds, and the second two stages equated to jogging/running speeds. Capillary blood samples were taken pre-, midway (at minute 8) and immediately post- the submaximal exercise test. Participants then rested for 1 h before completing a maximal exercise test. In the maximal test, the participants ran at a pre-determined individual fixed speed (inter-participant range 7 to 10 km·h^−1^) while the treadmill belt gradient was raised by 1% every minute (1 min stages) until volitional termination was attained. Finger-prick capillary blood samples were taken immediately pre- and post- the maximal exercise test. Expired air samples were collected during at least the last three one-minute stages of the maximal exercise test using standard Douglas bag methods to measure peak oxygen consumption ($\dot{V}O$_2_ peak). The study protocol is presented in Figure 1.

### 2.3. Anthropometry and Physical Maturity

Stature was measured using a stadiometer (The Leicester height measure, Seca Ltd., Birmingham, UK) to the nearest 0.01 m. Body mass (BM) was measured, and percentage body fat was estimated using bioelectrical impedance (Tanita BC-418MA, Tanita Corporation, Tokyo, Japan) to the nearest 0.1 kg and 0.1%, respectively, while participants stood barefoot wearing light clothes. Body mass index (BMI) was calculated by dividing the body mass (kg) by the stature squared (m^2^). Consequently, weight status was determined using age and sex-specific BMI cut off points [26]. Waist circumference was taken from the central point between the 10th rib and the iliac crest using a non-flexible tape measure [27]. Physical maturity was estimated through a self-assessment of secondary sexual characteristics. The scale ranges from 1 (prepubescent) to 5 (adult) [28].

### 2.4. Blood Sampling and Analyses

To enhance blood flow to the hand, the participants were asked to immerse their whole hand into a hot water (40 °C) container for 5 min, after which the hand was dried immediately, and a finger was cleaned with an alcohol swab and then pricked with a lancet (Unistick 3 Extra, Owen Mumford, UK). The first drop of blood was wiped, and 300 to 600 μL of blood was drawn into microvette tubes (Sarstedt Ltd., Leicester, UK). The sample tubes were placed immediately into a centrifuge at 12,800× *g* for 15 min (Eppendorf 5415c, Hamburg, Germany) to allow collection and storage of the resulting plasma at −80 °C for subsequent batch analysis. Plasma glucose concentration was analysed using a benchtop analyser (Pentra 400; HORIBA ABX Diagnostics, Montpellier, France) using enzymatic, colourimetric methods (HORIBA ABX Diagnostics). Further analyses of fasting plasma samples were completed to determine insulin concentrations using an enzyme-linked immunosorbent assay (Mercodia AB, Uppsala, Sweden). The intra-assay coefficients of variation for the duplicate samples were 0.7% for plasma glucose and 4.4% for plasma insulin. Fasting plasma glucose and insulin concentrations were used to calculate the homeostatic model assessment of insulin resistance (HOMA-IR) [29].

### 2.5. FGM Accuracy Assessments and Statistical Analyses

The scanned [ISFG] were paired with [CPG] measurements at 10 time points. The glucose data were divided into three segments representing three distinct events (i.e., during OGTT (5 time points), submaximal exercise test (3 time points) and maximal exercise test (2 time points)) to evaluate [ISFG] and [CPG] agreement to feeding and exercise independently. Matched glucose values during OGTT (i.e., at 5 time points, including fasting) were used to calculate total (tAUC) and incremental (iAUC) areas under the curve, peak glucose and time to peak using a time series response analyser [30]. Several methods were performed to assess the sensor accuracy of FGM, as there are no universally agreed standards to assess CGM accuracy [31].

The mean absolute (non-directional) relative difference (MARD) of the pooled glucose measurements at segmented events and at individual time-points were calculated using the following formula: (|ISFG − CPG|)/CPG × 100. Additionally, individual MARD was calculated using all paired glucose for each individual to examine the inter-individual variation. Correlation is described using Pearson’s correlation coefficient. The magnitude of effect sizes for the correlation coefficients of 0.10, 0.30 and 0.50 are small, medium, and large, respectively [32]. The percentage of paired results fulfilling the International Organization for Standardization (ISO) 15197:2013 criteria (mainly developed for glucometers) was assessed. The ISO 15197:2013 requires (1) 95% of results from the device to fall within ±0.83  mmol∙L^−1^ (15  mg∙dL^−1^) when reference glucose <5.56  mmol∙L^−1^ (100 mg∙dL^−1^) or within ±15% when reference glucose values ≥5.56  mmol∙L^−1^; (2) at least 99% of results have to be within zones A and B in the Consensus Error Grid (CEG) (also known as the Parkes Error Grid) [33]. Bland–Altman 95% (ratio) limits of agreement (LoA) were used to assess the agreement between [ISFG] and [CPG] for the segmented events. The maximal exercise glucose samples were transformed using a natural logarithm (log_e_) due to significant or meaningful heteroscedastic random errors; thus, 95% ratio LoA were calculated.

Linear mixed models repeated for site and time points were used to examine differences between [ISFG] and [CPG] during the three distinct events. The differences between tAUC, iAUC, peak glucose and time to peak during OGTT were examined using linear mixed models repeated for the site ([ISFG] and [CPG]). OGTT glucose, iAUC, peak glucose and time to peak data were log_e_ transformed due to non-normal residuals. These data are presented as geometric mean (95% confidence interval [CI]), and analyses are based on ratios of geometric means and 95% CI for ratios. The effect size (d) was calculated to describe the magnitude of difference between glucose measurements according to the following thresholds: trivial (˂0.2), small (≥0.2), moderate (≥0.5) and large (≥0.8) [32]. Statistical analyses were completed using SPSS (version 25.0; SPSS Inc., Chicago, IL). Values are expressed as mean ± SD unless stated otherwise, and statistical significance was accepted at *p* ˂ 0.05.

## 3. Results

Nineteen healthy participants were recruited, and all completed the study. Two participants were excluded because they did not fast. Of the remaining seventeen participants, one did not complete the submaximal and maximal exercise test due to illness. Thus, the accuracy analyses included 17 participants during OGTT (i.e., 85 glucose pairs) and 16 participants during submaximal (i.e., 48 glucose pairs) and maximal exercise tests (i.e., 32 glucose pairs). The participants’ characteristics are presented in Table 1. The mean age was 12.8 ± 0.9 years, and nine were girls. One participant was overweight, and two participants were thin (grade 1) according to age and sex-specific BMI cut off points [26]. Four participants were classified as ‘at risk’ according to age-, sex-, and BMI-specific percentiles of HOMA-IR [34].

### FGM Accuracy

**Figure 2:**
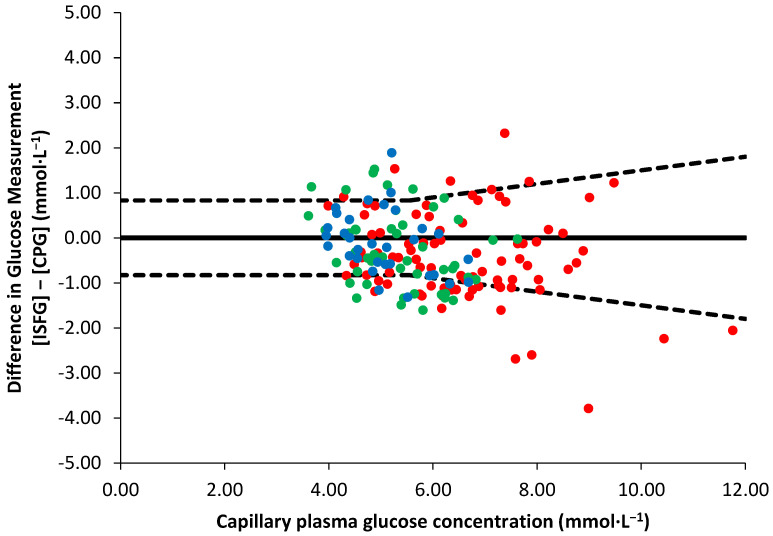
Scatter plot showing the differences in measurements between interstitial fluid glucose concentration ([ISFG]) obtained by a Flash glucose monitor and capillary plasma glucose concentration ([CPG]) during oral glucose tolerance test (red dots), submaximal (green dots) and maximal exercise (blue dots) tests. Dashed lines depict the accuracy boundaries applied based on ISO 15197:2013.

Overall, 68% (*n* = 113) of the paired glucose data met the ISO 15197:2013 criteria and were within the acceptable range, while the rest (32%, *n* = 52) were outside the set boundaries (Figure 2). The MARD of the 68% of glucose data meeting the ISO 15197:2013 criteria was 8.6 ± 4.9% and 23.0 ± 5.8% for the 32% that did not meet the ISO 15197:2013 criteria. The overall MARD was 13.1 ± 8.5%. The individual MARDs showed large inter-individual variations that ranged between 9.4% and 18.2%. Individual MARDs did not correlate with any participant characteristics, including age, BMI, %body fat, waist circumference, maturity status and $\dot{V}O$_2_ peak (*p* ≥ 0.150), nor did they differ between boys and girls (*p* = 0.380); it was not possible to identify an explicit cause for the inter-individual variability.

**Figure 3:**
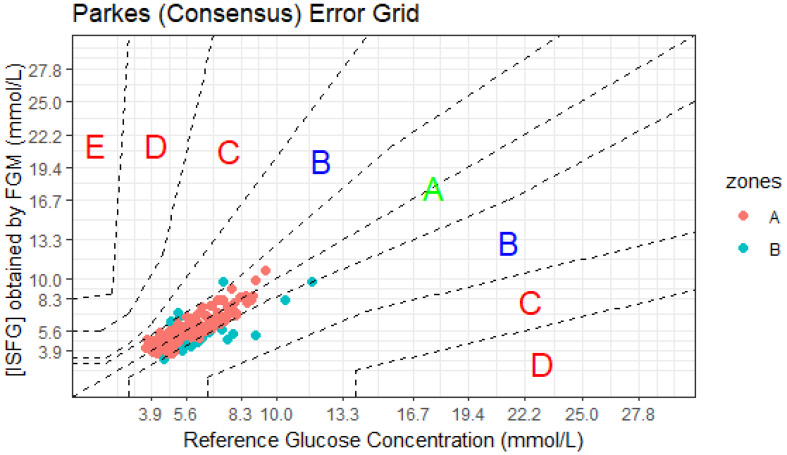
Consensus error grid (CEG) analysis of Flash glucose monitor (FGM). A total of 165 glucose pairs of [ISFG] obtained by an FGM and capillary plasma glucose concentration (reference method) were plotted in the CEG. According to ISO 15197:2013, 99% of measurement results shall be within CEG zones A and B. The FGM shows 100% of results within CEG zones A and B. Dashed lines are the boundaries of the different zones, implying different degrees of risk posed by inaccurate measurement. Zone A—no effect on clinical action; zone B—altered clinical action with little or no effect on clinical outcome; zone C—altered clinical action and likely to affect clinical outcome; zone D—altered clinical action which could have significant medical risk; zone E—altered clinical action, could have dangerous consequences.

When examining the clinical accuracy of FGM on the CEG (Figure 3), 84% (*n* = 139) and 16% (*n* = 26) of the FGM readings were found in zone A (which were classified as clinically accurate measurements) and zone B (altered clinical action, little or no effect on the clinical outcome), respectively, which met the ISO 15197:2013 criteria of having at least 99% of results within zones A and B. These results indicate that the deviated readings of FGM from the reference method (i.e., sensor error) would not severely impact clinical decisions.

**Figure 4:**
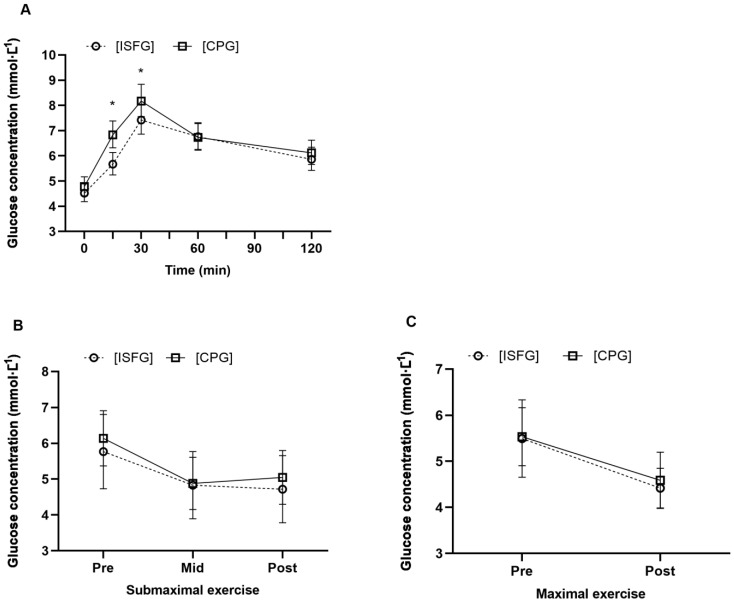
Changes in interstitial fluid glucose ([ISFG], open circle) and capillary plasma glucose ([CPG], open square) concentrations during an oral glucose tolerance test (**A**), submaximal (**B**) and maximal (**C**) exercise tests. Data points for oral glucose tolerance test (**A**) represent geometric mean and corresponding 95% confidence interval as error bars from *n* = 17 participants, and the statistical analyses are based on natural log transformed data. Data points for submaximal (**B**) and maximal (**C**) exercise tests represent mean and standard deviation as error bars from *n* = 16 participants. * Mean [ISFG] was significantly lower than [CPG] at 15 (*p* < 0.001) and 30 min (*p* = 0.041) after glucose loading. No significant differences were found between [ISFG] and [CPG] at each time point during submaximal and maximal exercise tests.

The differences between the glucose measurements of the two methods are presented in Table 2. Overall, FGM provided lower [ISFG] on average than [CPG] during OGTT, with a small effect size (*p* < 0.001, d = 0.35). While post-OGTT [ISFG] responses resulted in 0.28 mmol∙L^−1^ (~4%) lower tAUC and 0.10 mmol∙L^−1^ (~5%) lower iAUC on average than [CPG], the differences were not statistically significant, with small and trivial effect sizes, respectively. Pairwise comparisons between [ISFG] and [CPG] at each time point revealed significantly large and moderate differences at 15 and 30 min, respectively (Table 2 and Figure 4A). Peak [ISFG] was lower by 0.54 mmol∙L^−1^ (~7%) compared to [CPG] during OGTT, and the mean time to peak for [ISFG] was 37 min (95% CI 31 to 43 min) and 30 min (95% CI 25 to 35 min) for [CPG], indicating a lag time of 7 min on average. Non-significant site by timepoint interactions were observed during the exercise tests (*p* ≥ 0.614), indicating that the pattern of [ISFG] was similar to [CPG] across the time points (Figure 4B,C).

There were large, positive correlations between paired [ISFG] and [CPG] values during the OGTT (r = 0.789, 95% CI 0.69 to 0.86, r^2^ = 62%, *p* < 0.001) and the submaximal (r = 0.646, 95% CI 0.44 to 0.79, r^2^ = 42%, *p* < 0.001) and maximal (r = 0.622, 95% CI 0.35 to 0.80, r^2^ = 39%, *p* < 0.001) exercise tests. Bland–Altman plots showed lower [ISFG] than [CPG] (i.e., negative biases) during the OGTT (−0.47 mmol∙L^−1^, *p* < 0.001; 95% LoA, −2.40 to 1.54 mmol∙L^−1^) and submaximal exercise (−0.25 mmol∙L^−1^, *p* = 0.046; 95% LoA, −1.92 to 1.42 mmol∙L^−1^), but not during the maximal exercise test (0.98, *p* = 0.326; 95% ratio LoA, 0.75 to 1.27).

## 4. Discussion

This study was the first to assess the performance of the FreeStyle Libre flash glucose monitoring system in healthy adolescents in response to feeding and exercise. The overall MARD versus [CPG] reference values was 13.1 ± 8.5%, consistent with previous studies assessing the same glucose monitoring system in young people [12,18,19] and adults [35,36] living with T1D. The performance of FGM was previously assessed against capillary blood glucose in 87 young people aged 4–17 years with T1D under free-living conditions, and it resulted in an overall MARD of 13.9% [12]. Likewise, results from 78 adolescents aged 11–15 years with T1D reported a slightly higher MARD than ours against a capillary blood glucose of 13.5% during a summer camp, reflecting comparable accuracy during a free-living setting that included physical activities [19]. Moreover, the sensor performance was examined under controlled condition simulating real-life events (e.g., meals, exercise, hypo-, and hyperglycaemia) against venous plasma glucose concentrations and reported an overall MARD of 13.2% in adults with T1D [36]. It is worth noting that the blood sources that the FGM is being compared with (as capillary blood) has shown to be more sensitive to change than venous samples [37]. Nevertheless, these results indicate similar sensor accuracy in healthy young people, despite greater glycaemic variability in people living with T1D [12,18,19].

An OGTT was used to induce a large glycaemic excursion and to assess the magnitude of sensor bias from the laboratory-based reference method used in our paediatric research. The [ISFG] largely correlated with [CPG] during the OGTT (r^2^ = 62%, *p* < 0.001), and the MARD during the standardised OGTT was 13.4 ± 5.0%. However, FGM demonstrated a mean bias of −0.47 mmol∙L^−1^ across all glucose measurements during OGTT compared with the [CPG] reference method. Specifically, the differences between [ISFG] and [CPG] were statistically significant with large and moderate effect sizes, respectively, after 15 (−1.16 mmol∙L^−1^) and 30 min (−0.75 mmol∙L^−1^) of the OGTT. The large mean difference at 15 min coincides with the greatest residual assessed, with a MARD of 17.5%. The sensor discrepancy was reported to increase up to a MARD of 17% when glucose increased rapidly by more than 0.08 mmol∙L^−1^∙min^−1^ [38]. Similarly, in a recent study in healthy adults with obesity, MARDs were found to be the highest (~25%) 15 and 30 min after consuming a standard breakfast [21]. After consuming an OGTT, glucose absorption in the intestine increased glucose appearance in blood circulation, with the peak value at 30 min. Yet, glucose concentration in the interstitial fluid compartment did not match this rise, most likely due to the time required for the glucose to equilibrate in the two compartments (known as the physiological lag) [39,40]. The FGM estimates blood glucose concentration by measuring [ISFG] using algorithms [15]. Although the [ISFG] peak value lagged by 7 min on average, the mean peak [ISFG] was significantly lower than [CPG] by ~7% (−0.54 mmol∙L^−1^, d = 0.52), indicating that both physiological lag and systematic bias may have affected the sensor accuracy [41]. Nevertheless, total (tAUC) and incremental (iAUC) glucose area under the curves, which are commonly used postprandial glycaemic outcomes, were not significantly different between the two glucose measurement methods. It is worth noting that a lack of statistically significant differences may not necessarily mean equivalence of measurement, yet the magnitude of the difference was trivial to small. A similar study in healthy adults without diabetes showed that the differences from pre-meal baseline were not significantly different between [ISFG] and [CPG] at all time points, except at 15 and 30 min after breakfast consumption [20]. In addition, a study in healthy children (9.9 ± 1.4 years) without diabetes using the professional version of FreeStyle Libre has shown comparable glucose AUC values after an OGTT when compared with intravenously obtained plasma glucose values [9]. These results indicate that FGM is an acceptable device, reflecting postprandial glycaemic responses that have high relevance to cardiometabolic disease risk [1].

During exercise, glucose concentration in the interstitial fluid may decrease before plasma glucose due to increased glucose uptake in the exercising muscles; therefore, some difference in [ISFG] versus [CPG] would be expected. The FGM read slightly lower than [CPG] (by 0.25 mmol∙L^−1^ on average) during submaximal exercise (three time points), but not during maximal exercise (two time points). The [ISFG] was similar to [CPG] across the five glucose sampling time points taken during the exercise tests. The combined MARD for mid- and post-submaximal exercise was 15.4%. Similarly, MARD values (16.2 to 19.1%) have been seen when comparing FGM and plasma glucose concentrations during different walking conditions in healthy adults with obesity [21]. Due to the difficulty of obtaining finger-prick blood samples during maximal exercise, blood samples were taken after participants reached the exhaustion point when the body was under high metabolic stress. The findings of our study showed a low (i.e., better accuracy) MARD result of 8.8 ± 6.4% compared to the [CPG] reference values after the maximal exercise test. In contrast, a recent study with healthy adults reported reduced sensor accuracy (median ARD of 16.2% with high values in the clinically unsafe zone) when FGM was compared with a glucometer during high intensity intermittent (not maximal) exercise performed 2 h after consuming a carbohydrate-rich breakfast [22]. That said, studies examining the accuracy of FreeStyle Libre during different exercise intensities remain rare. Therefore, further research with a larger number of glucose pairs is needed to confirm the effect of exercise on sensor accuracy in different populations.

The magnitude of the differences between the two glucose measurement methods was larger during OGTT (7%) than during submaximal (5%) and maximal (2%) exercise tests. Two possible reasons for these differences are (1) the rate of glycaemic change after an OGTT may be higher than that during or after an exercise bout of short duration, and/or (2) the sampling frequency was higher during OGTT than exercise tests. Regarding Bland–Altman analyses, FGM underestimated the laboratory-based reference method for measuring [CPG] during OGTT and submaximal but not maximal exercise tests. Yet, the 95% LoA were wide under each condition, which is consistent with other studies assessing the same glucose monitoring system [36,42] or other continuous glucose monitoring systems [43,44,45]. Unlike blood glucose monitoring systems, there are no universally agreed standards for assessing CGM accuracy [31]. Using the ISO 15197:2013 criteria, our results showed that only 68% (*n* = 113) of the paired glucose data met the ISO 15197:2013 criteria and were within the acceptable range. Although this criterion was developed to define the accuracy and precision requirements necessary for blood glucose monitoring systems (also known as glucometer devices), King et al. (2018) reported that ~46% of the blood glucose monitoring systems currently available on the market failed to meet ISO 15197:2013 [46]. Nevertheless, the Consensus Error Grids (CEG) analyses revealed that all FGM readings were found in zone A (84%, *n* = 139) or B (16%, *n* = 26), indicating that results from FGM are clinically acceptable for making treatment decisions. These results align with other studies [20,21,23].

Although large inter-individual variability in the sensor accuracy was found in the present study, the individual factors assessed (e.g., age, sex, BMI, %body fat, waist circumference, maturity status and $\dot{V}O$_2_ peak) did not appear to explain this variation. In support, some studies have shown that sensor accuracy is independent of individual characteristics such as age, sex, body mass or BMI in adults [24] or young people living with T1D [12]. However, other studies using the professional FreeStyle Libre found that sensor accuracy was lower in overweight/obese children without diabetes compared with normal weight children [9]. Our sample did not vary sufficiently in weight status to be able to identify such a correlation. Therefore, a larger sample size that includes healthy young people with heterogeneous weight status is required in future studies to address this specifically. The glucose monitor was assessed in a single day (i.e., day 2 or 3 of sensor wear), that is, after 24 h of sensor wear, which was deemed to be the least accurate across the 14 wearing days [24,47]. Therefore, its accuracy during the remaining days is not known. Yet, previous studies have shown stable sensor performance across 14 days apart from the insertion day [24,47].

A strength of the current study is that glucose concentrations were measured during a number of different conditions (i.e., postprandially, at rest and during various exercise intensities). However, a limitation of this was that the number of finger-prick blood samples for any given condition or time period was restricted, as it was not felt appropriate to subject the adolescent participants in this study to more frequent sampling. This precluded the determination of the rate of change in glucose concentration and the effect this had on the sensor performance. Further research should be undertaken to investigate this important issue.

## 5. Conclusions

The FreeStyle Libre Flash glucose monitoring system was found to be a feasible alternative to finger prick methods when used to examine postprandial glycaemic responses in healthy adolescents (an under-researched group), with small to trivial differences between methods for tAUC and iAUC. Use of FGM during exercise was also found to be acceptable, with only a small difference between methods found and with FGM concentrations slightly lower than those of finger prick methods during submaximal exercise but not maximal exercise (most studies to date have examined the performance of the devices at rest). Care may need to be taken when using FGM during periods of rapid change in glucose concentrations, as FGM glucose concentrations were found to be lower after 15 and 30 min of the OGTT than finger prick methods.

## Figures and Tables

**Table 1:** Participant characteristics (*n* = 17).

Variable	Mean ± SD	Range
Age (y)	12.8 ± 0.9	11.5–14.4
Stature (m)	1.56 ± 0.1	1.35–1.75
Body mass (kg)	44.7 ± 7.2	30.3–56.9
BMI (kg∙m^−2^)	18.4 ± 2.1	15.4–22.1
Body fat (%)	22.2 ± 4.9	12.7–31.5
Waist circumference (cm)	60.3 ± 8.9	46.0–72.0
Breast development *	4 (1)	1–4
Genital development *	2 (1)	1–3
Pubic hair development *	3 (2)	1–5
HOMA-IR	1.62 ± 1.01	0.44–3.74
$\dot{V}O$_2_ peak (mL∙kg^−1^∙min^−1^) **	42.3 ± 10.1	31.6–58.8

BMI, body mass index; $\dot{V}O_{2}$, oxygen consumption; HOMA-IR, homeostatic model assessment of insulin resistance. * Self-assessment—median (interquartile range). Two participants did not complete the assessment (*n* = 15). ** One participant did not complete both exercise tests, and maximal exercise test data were not available for another participant due to a technical error (*n* = 15).

**Table 2:** Differences between [ISFG] obtained by FGM and [CPG] at each time point during OGTT (*n* = 17), submaximal and maximal exercise tests (*n* = 16).

	[ISFG] (mmol∙L^−1^) *	[CPG] (mmol∙L^−1^) *	Mean Difference (95% CI) *	MARD (%)	*p*-Value	Effect Size
Overall OGTT	5.96 (5.65 to 6.29)	6.43 (6.09 to 6.78)	−7.3% (−11 to −3%)	13.4 ± 5.0	***<0.001***	***0.35***
0 min	4.52 (4.18 to 4.89)	4.78 (4.42 to 5.17)	−5.4% (−14 to 4%)	14.1 ± 6.3	0.240	0.57
15 min	5.67 (5.24 to 6.13)	6.83 (6.32 to 7.39)	−17.1% (−24 to −9%)	17.5 ± 11.2	***<0.001***	***1.21***
30 min	7.42 (6.86 to 8.02)	8.17 (7.56 to 8.84)	−9.2% (−17 to 0%)	10.6 ± 6.1	***0.041***	***0.55***
60 min	6.76 (6.25 to 7.31)	6.73 (6.22 to 7.28)	0.5% (−8 to 10%)	13.4 ± 7.4	0.923	0.17
120 min	5.86 (5.42 to 6.34)	6.12 (5.66 to 6.62)	−4.2% (−13 to 5%)	11.4 ± 7.5	0.365	0.29
Glucose iAUC	1.79 (1.47 to 2.20)	1.89 (1.54 to 2.31)	−4.9% (−19 to 12%)	22.1 ± 15.1	0.529	0.14
Glucose tAUC	6.52 ± 1.06	6.80 ± 0.64	−0.28 (−0.61 to 0.04)	8.5 ± 5.8	0.084	0.45
Peak glucose	7.81 (7.24 to 8.43)	8.35 (7.74 to 9.01)	−6.5% (−12 to −1%)	11.0 ± 5.6	***0.032***	***0.52***
Overall submaximal exercise	5.11 ± 1.06	5.36 ± 0.93	−0.25 (−0.54 to 0.04)	14.0 ± 4.4	0.093	0.27
Pre submaximal exercise	5.77 ± 1.04	6.14 ± 0.76	−0.37 (−0.88 to 0.14)	11.4 ± 7.7	0.149	0.49
Mid submaximal exercise	4.83 ± 0.94	4.88 ± 0.73	−0.05 (−0.55 to 0.46)	14.6 ± 9.4	0.859	0.07
Post submaximal exercise	4.72 ± 0.94	5.05 ± 0.75	−0.34 (−0.84 to 0.17)	16.1 ± 9.0	0.193	0.44
Overall maximal exercise	4.96 ± 0.85	5.06 ± 0.78	−0.11 (−0.38 to 0.17)	11.0 ± 5.0	0.441	0.14
Pre maximal exercise	5.49 ± 0.84	5.54 ± 0.63	−0.04 (−0.43 to 0.35)	13.2 ± 8.5	0.830	0.08
Post maximal exercise	4.42 ± 0.43	4.59 ± 0.61	−0.17 (−0.56 to 0.22)	8.8 ± 6.4	0.382	0.64

[ISFG], interstitial fluid glucose concentration; FGM, Flash glucose monitor; [CPG], capillary plasma glucose concentration; OGTT, oral glucose tolerance test; iAUC, incremental area under curve; tAUC, total area under curve; MARD, mean absolute relative difference. Mean differences between [ISFG] and [CPG] were examined using linear mixed models. Post-hoc pairwise comparisons were examined with the Holm–Bonferroni correction for multiple comparisons. A *p*-value < 0.05 depicts statistical significance. * For log transformed data, values are presented as geometric means, and corresponding 95% CI and pairwise comparisons are presented as percentage difference (%) based on ratios of geometric means and corresponding 95% CI (%). For untransformed (normally distributed) data, values are expressed as mean ± standard deviation (SD), and pairwise comparisons are presented as mean absolute difference and corresponding 95% CI.

## Data Availability

The data presented in this study are available on request from the corresponding author. The data are not publicly available due to ethical reasons of patient data.
